# *Tanacetum* *polycephalum* (L.) Schultz-Bip. Induces Mitochondrial-Mediated Apoptosis and Inhibits Migration and Invasion in MCF7 Cells

**DOI:** 10.3390/molecules19079478

**Published:** 2014-07-03

**Authors:** Hamed Karimian, Syam Mohan, Soheil Zorofchian Moghadamtousi, Mehran Fadaeinasab, Mahboubeh Razavi, Aditya Arya, Behnam Kamalidehghan, Hapipah Mohd Ali, Mohamad Ibrahim Noordin

**Affiliations:** 1 Department of Pharmacy, Faculty of Medicine, University of Malaya, Kuala Lumpur 50603, Malaysia; E-Mails: mahbobehrazavi@gmail.com (M.R.); aditya@um.edu.my (A.A.); behnam@um.edu.my (B.K.); ibrahimn@um.edu.my (M.I.N.); 2 Medical Research Centre, Jazan University, Jazan 2092, Saudi Arabia; E-Mails: syammohanm@yahoo.com (S.M.); hapipah@um.edu.my (H.M.A.); 3 Biomolecular Research Group, Biochemistry Program, Institute of Biological Sciences, Faculty of Science, University of Malaya, Kuala Lumpur 50603, Malaysia; E-Mail: soheil.zorofchian@gmail.com; 4 Department of Chemistry, Faculty of Science, University of Malaya, Kuala Lumpur 50603, Malaysia; E-Mail: mehranfadaie_n@yahoo.com

**Keywords:** *Tanacetumpolycephalum*, breast cancer, apoptosis, MCF7 cell line, cell cycle, Bax/Bcl-2, immunofluorescent, RT-PCR

## Abstract

*Tanacetum* *polycephalum* (L.) Schultz-Bip (Mokhaleseh) has been traditionally used in the treatment of headaches, migraines, hyperlipidemia and diabetes. The present study aimed to evaluate its anticancer properties and possible mechanism of action using MCF7 as an *in* *vitro* model. *T.* *polycephalum* leaves were extracted using hexane, chloroform and methanol solvents and the cytotoxicity was evaluated using the MTT assay. Detection of the early apoptotic cells was investigated using acridine orange/propidium iodide staining. An Annexin-V-FITC assay was carried out to observe the phosphatidylserine externalization as a marker for apoptotic cells. High content screening was applied to analyze the cell membrane permeability, nuclear condensation, mitochondrial membrane potential (MMP) and cytochrome *c* release. Apoptosis was confirmed by using caspase-8, caspase-9 and DNA laddering assays. In addition, Bax/Bcl-2 expressions and cell cycle arrest also have been investigated. MTT assay revealed significant cytotoxicity of *T.* *Polycephalum* hexane extract (TPHE) on MCF7 cells with the IC_50_ value of 6.42 ± 0.35 µg/mL. Significant increase in chromatin condensation was also observed via fluorescence analysis. Treatment of MCF7 cells with TPHE encouraged apoptosis through reduction of MMP by down-regulation of Bcl-2 and up-regulation of Bax, triggering the cytochrome *c* leakage from mitochondria to the cytosol. The treated MCF7 cells significantly arrested at G_1_ phase. The chromatographic analysis elicited that the major active compound in this extract is 8β-hydroxy-4β,15-dihydrozaluzanin C. Taken together, the results presented in this study demonstrated that the hexane extract of *T.* *Polycephalum* inhibits the proliferation of MCF7 cells, resulting in the cell cycle arrest and apoptosis, which was explained to be through the mitochondrial pathway.

## 1. Introduction

Cancer is the second most prevalent cause of death in economically developing countries and a leading cause of death in developed countries [1]. About 13.7 million Americans with cancer history were alive in the beginning of 2012, and this number will rise to roughly 18 million by 2022 [2,3]. Amongst the various types of cancer, breast cancer is the most frequently diagnosed cancer (41%) in American women. An estimated three million cases of invasive breast cancer have been reported among women in the US during 2012 [2,3].

Over the past several decades, significant efforts have been made in the scientific and commercial area for the discovery of new anticancer drugs [4]. The costs of cancer therapy by chemical and synthetic drugs are very high and still there is no extremely effective drug for treatment of most cancers [5,6]. Thus, the need for development of natural medicines has been high [7]. The effects of fruits and vegetables on the prevention and reduction in cancer risk are well known and it has been proved that plants are one of the crucial sources of anticancer drugs [8]. Natural products’ contribution to cancer therapy is more evident considering the fact that they were involved in the development of roughly 75% of novel anticancer agents between 1981 and 2010 [9]. Thereby, isolation and characterization of natural products with anticancer activity is one of the most important aspects to be considered.

*Tanacetum* *polycephalum* (L.) Schultz-Bip (Mokhaleseh) belonging to the family of Asteraceae is an aromatic perennial plant which grows mostly in Iran, Iraq and Turkey [10,11]. Members of this family with more than 1,600 genera and 2,300 species have been subjected to various scientific inspections due to their extensive biological activities [10,12]. Previous studies on *T. polycephalum* (L.) Schultz-Bip were mostly limited to the composition of the essential oils isolated from this species [11,13,14,15]. However, antiallergic, anticancer, anti-irritant, antiseptic, anesthetic, analgesic, disinfective and expectorant properties are mentioned for this plant [15]. Other species in *Tanacetum* genera, including *T. gracile* and *T. parthenium* have been proved to be cytotoxic against various cancer cells [16,17]. Through the previous studies, the active compounds of *Tanacetum* species with apoptotic effects have been investigated, such as parthenolide, which induces apoptosis in acute myelogenous leukemia (AML) cells and leaves normal bone marrow cells relatively unscathed [18,19,20,21]. Considering the anticancer potential of plants in *Tanacetum* genera, in the present study for the first time, the anticancer activity of *Tanacetum* *polycephalum* (L.) Schultz-Bip extract against MCF7 human breast cancer cell line and its possible mechanisms of action have been investigated.

## 2. Results and Discussion

### 2.1. Antiproliferative Effect of T. Polycephalum Hexane Extract (TPHE) on MCF7 Cells

**Figure 1:**
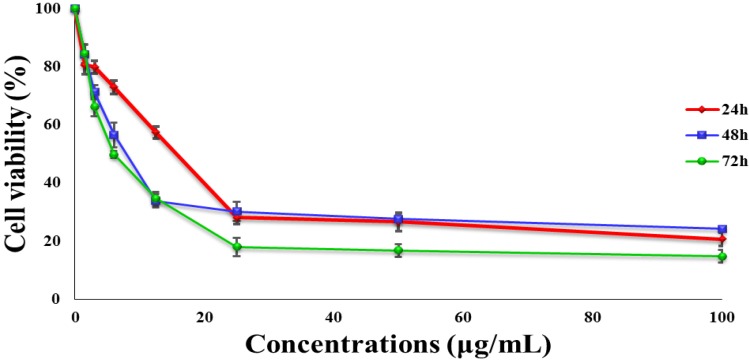
The tested agent induced cell cytotoxicity on MCF7 cells in a time-dependent manner. The IC_50_ value of TPHE at 24, 48 and 72 h on the MCF7 cell line was determined to be 24.65 ± 2.41, 6.42 ± 0.35 and 5.16 ± 1.6 μg/mL, respectively. The data are shown as the mean ± SD (*n* = 3).

The cytotoxic effect of TPHE on various cell lines was examined by the MTT assay. The assay results demonstrated that TPHE had different degrees of antiproliferative activity on cancer and normal cell lines, with IC_50_ values ranging from 6.42 ± 0.35 to 100 ± 3.5 µg/mL after 48 h of treatment (Table 1). Meanwhile, chloroform and methanol extracts indicated no significant anti-proliferative effect towards cancer cells, compared to TPHE (Table 1). Amongst the tested cell lines, MCF7 cells were found to be the most sensitive cells to TPHE in a concentration and time-dependent manner with the IC_50_ value of 6.42 ± 0.35 µg/mL (Figure 1), while the positive control of tamoxifen showed the IC_50_ value of 1.5 ± 0.15 µg/mL towards MCF7 cells. In addition, TPHE did not show any noteworthy signs of toxicity on the normal cell lines CD841 and WRL-68. DMSO (0.1%) which was used as a vehicle control did not show any sign of toxicity.

**Table 1:** IC_50_ values of *T. polycephalum* leaves extracts on nine different cell lines after 48 h treatment.

Cell Lines	IC_50_ (μg/mL)		
	Hexane	Chloroform	Methanol
MCF7	6.42 ± 0.35	45.66 ± 6.43	79.42 ± 7.43
CEMss	7.22 ± 0.94	54.54 ± 4.8	66.52 ± 5.32
MDA-MB-231	7.83 ± 1.34	76.43 ± 5.24	77.52 ± 8.34
PC3	10.11 ± 1.16	65.31 ± 3.69	68.32 ± 4.54
HT29	13.43 ± 1.67	88.29 ± 7.5	80.13 ± 6.61
HepG2	18.46 ± 2.21	90.81 ± 2.43	95.59 ± 2.43
A549	22.39 ± 3.6	72.85 ± 6.83	98.31 ± 2.38
CCD841	76.73 ± 4.72	98.9 ± 6.68	99.26 ± 4.34
WRL-68	100 ± 3.5	95.28 ± 4.28	98.72 ± 6.54

The data are shown as the mean ± SD (*n* = 3).

### 2.2. Gas Chromatography Profile of TPHE

**Figure 2:**
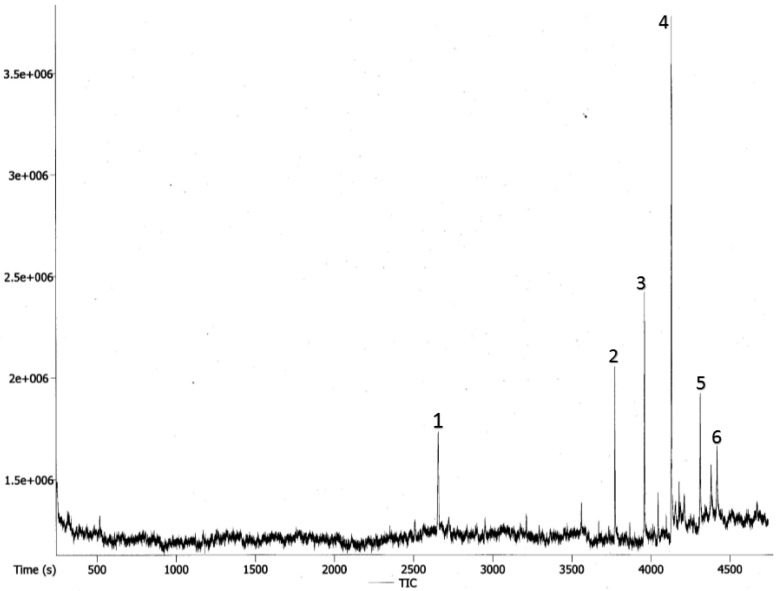
The chromatogram analysis of TPHE characterized with the GC-MS-TOF.

The hexane extract was characterized by GC-MS-TOF (Figure 2). The chromatographic analysis showed that the major sesquiterpene lactone compound in this fraction is 8β-hydroxy-4β,15-dihydro- zaluzanin C (Table 2).

**Table 2:** GC-MS-TOF analysis of the hexane extract.

Peak No.	Name of Compounds	Retention Time (s)	Mass
1	Z-Isocitral	2654.45	152
2	Tetracosane	3771.2	338
3	Hexacosane	3957.15	366
4	8β-hydroxy-4β,15-dihydrozaluzanin C	4127.4	264
5	Octacosane	4309.5	394
6	Hentriacontane	4415.95	436

### 2.3. Quantification of Apoptosis Induced by TPHE Using Acridine Orange (AO)/Propidium Iodide (PI) Double-Staining

**Figure 3:**
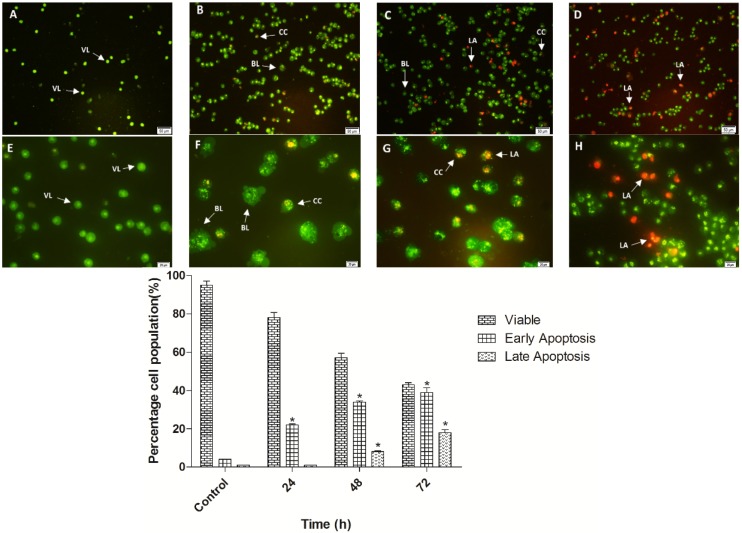
Apoptotic morphological changes of MCF7 cells were observed by fluorescent microscope (×20 and ×40). (**A**,**E**) Untreated MCF7 cells demonstrated normal structure without prominent apoptosis after 72 h. (**B**,**F**) Early apoptotic features, including chromatin condensation and cell blebbing were observed after 24 h represented by intercalated AO dye (bright green). (**C**,**G**), (**D**,**H**) After 48 and 72 h, clear hallmarks of late apoptosis namely, blebbing and fragmented DNA were detected by orange color of PI. BL: Membrane blebbing; CC: Chromatin condensation; LA: Late apoptosis; VI: Viable MCF7 cells. * represents significant difference (*p* < 0.05) compared with the control.

Morphological changes of MCF7 cells, including viability, early and late features of apoptosis were monitored under the fluorescence microscope after treatment with TPHE for 24, 48 and 72 h. After 24 h, MCF7 cells showed early apoptotic features, including chromatin condensation and membrane blebbing (Figure 3B,F). Chromatin condensation was detected by the intervention of AO within the condensed DNA shown by bright green fluorescence. Simultaneously, a green intact nuclear structure showed the healthy MCF7 cells (Figure 3A,E). After 48 and 72 h, PI binding to denatured DNA shown by reddish-orange color confirmed the late stage of apoptosis induced by TPHE (Figure 3C,G and 3D,H). The result depicted that TPHE induced morphological characterizations of apoptosis in a time-dependent manner.

### 2.4. Detection of Early Apoptosis Induced by TPHE Using Annexin-V-FITC Labeling

**Figure 4:**
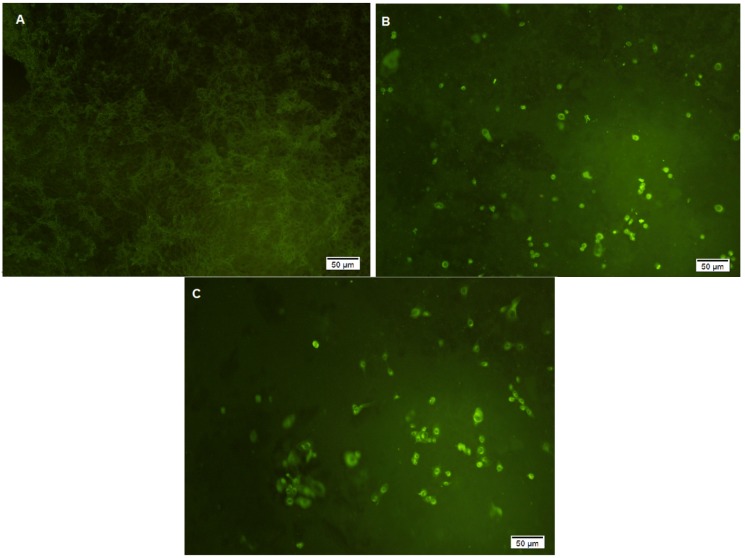
MCF7 cells were treated with IC_50_ concentration of TPHE for 24 h. (**A**) Untreated cells were shown as control indicating viable cells. After treatment, the translocation of PS residues to the outer membrane of stained cells with Annexin-V-FITC shown in light green proved the induction of early apoptosis observed using fluorescence microscope with (**B**) ×20 and (**C**) ×40 magnifications.

**Figure 5:**
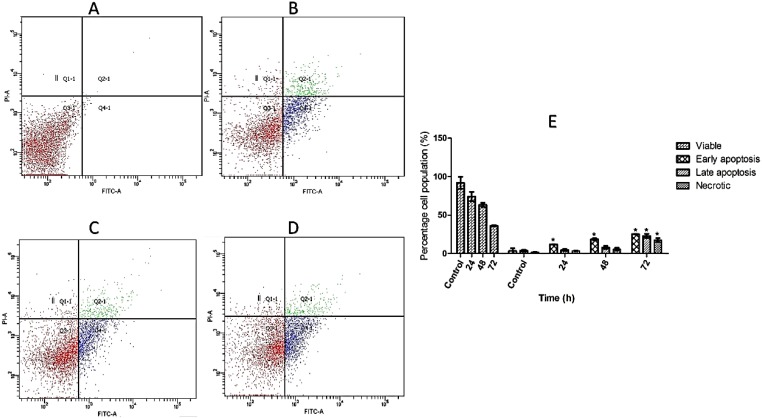
Time-dependent induction of early and late apoptosis by TPHE after (**B**) 24, (**C**) 48 and (**D**) 72 h of treatment. (**A**) represents the untreated cells as the control. (**E**) Representative bar chart depicted the percentage of early apoptotic, late apoptotic and necrotic cells. The data are shown as the mean ± SD (*n* = 3). * represents significant difference (*p* < 0.05) compared with the control.

The perturbation in the plasma membrane asymmetry because of phosphatidylserine (PS) externalization is considered one of the important markers for detection of early apoptosis [22]. The result of Annexin-V-FITC staining assay obtained from fluorescent microscope images are shown in Figure 4. Induction of early apoptosis in the MCF7 treated cells with TPHE was clearly detected by PS externalization. As shown in Figure 4B,C, the clear light green representing the attachment of Annexin-V-FITC to translocated PS suggested the induction of early apoptosis. Meanwhile, the untreated cells did not show any sign of PS externalization (Figure 4A). The flow cytometry analysis of Annexin-V externalization revealed the quantity of viable (Annexin-V^−^/PI^−^), early apoptotic (Annexin-V^+^/PI^−^), late apoptotic (Annexin-V^+^/PI^+^) and necrotic (Annexin-V^+^/PI^−^) cells. As shown in Figure 5, the number of cells that underwent early apoptosis increased after 24, 48 and 72 h of treatment with TPHE. After 48 and 72 h of treatment, the late apoptotic population was elevated to approximately 25%. The microscopic observation associated with fluorescent analysis of Annexin-V confirmed the induction of apoptosis by TPHE.

### 2.5. TPHE Arrested MCF7 Cells at G_1_ Phase

**Figure 6:**
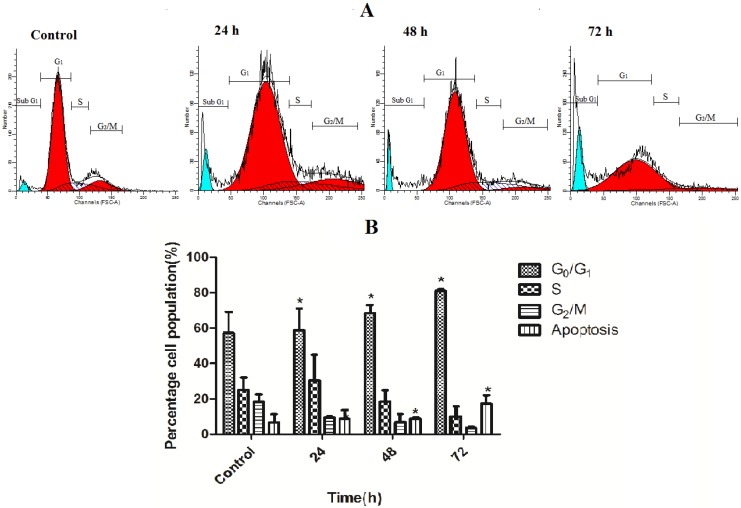
Effect of TPHE studied on cell cycle distribution of MCF7 cells. (**A**) After treatment with IC_50_ concentration of TPHE for 24, 48 and 72 h, flow cytometry was used to analyze the cell cycle. (**B**) The result demonstrated the cell cycle arrest at G_1_ phase. The percentage of apoptotic cells indicated the subG_1_ population after 24, 48 and 72 h treatment. The data are shown as the mean ± SD (*n* = 3). * represents significant difference (*p* < 0.05) compared with the control.

Flow cytometry analysis was carried out to investigate the effect of TPHE on the DNA content of MCF7 cells by cell cycle phase distribution (G_0_, G_1_, S, G_2_ and M) after treatment for 24, 48 and 72 h. The result of this experiment indicated that there is a significant G_1_ phase arrest in a time dependent manner, after 24, 48, and 72 h treatment (Figure 6). Meanwhile, the number of MCF7 cells in S and G_2_/M phases decreased after 72 h treatment. Additionally, the number of MCF7 cells in subG_1_ phase elevated after treatment by TPHE presenting the number of cells undergoing apoptosis.

### 2.6. THPE Activated Caspase-7, -8 and -9

**Figure 7:**
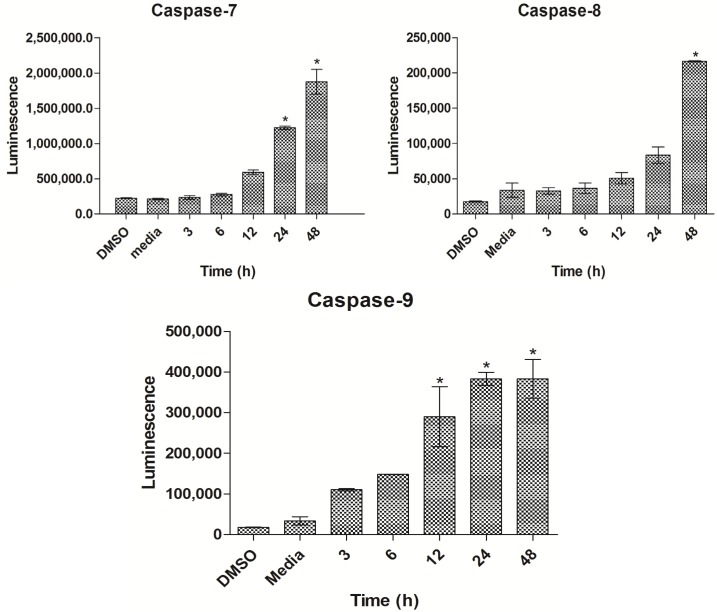
The luminescence assay demonstrated the activities of caspase-7, -8 and -9 on MCF7 cells treated with TPHE for 3, 6, 12, 24 and 48 h. The data are shown as the mean ± SD (*n* = 3). * represents significant difference (*p* < 0.05) compared with the control.

After treatment of MCF7 cells with IC_50_ concentration of TPHE for 3, 6, 12, 24 and 48 h, the caspase-7, -8 and -9 enzyme activities were determined. Caspase-8 activity was significantly increased in 48 h, while caspase-9 significantly increased in 12, 24 and 48 h of treatment (Figure 7). The executioner caspase-7 also showed significant elevation after 24 and 48 h.

### 2.7. THPE Activated Mitochondrial-Initiated Events

**Figure 8:**
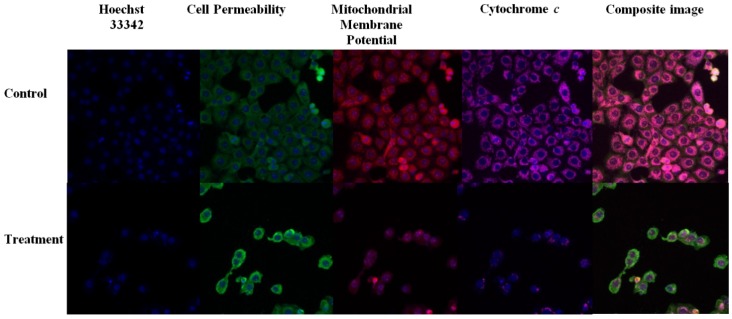
Representative images of MCF7 cells (×20) treated with TPHE at IC_50_ concentration. Cells stained with Hoechst 33342, cell membrane permeability, cytochrome *c* and MMP dyes. MCF7 cells produced a noteworthy reduction in MMP and a marked increase in chromatin condensation, cell membrane permeability and cytochrome *c* release.

To explore the apoptotic pathway induced by TPHE as confirmed in previous assays, we investigated critical apoptotic features in MCF7 cells. Hoechst 33342 staining depicted the chromatin condensation of MCF7 cells upon treatment with TPHE, shown in blue fluorescence (Figure 8). 

**Figure 9:**
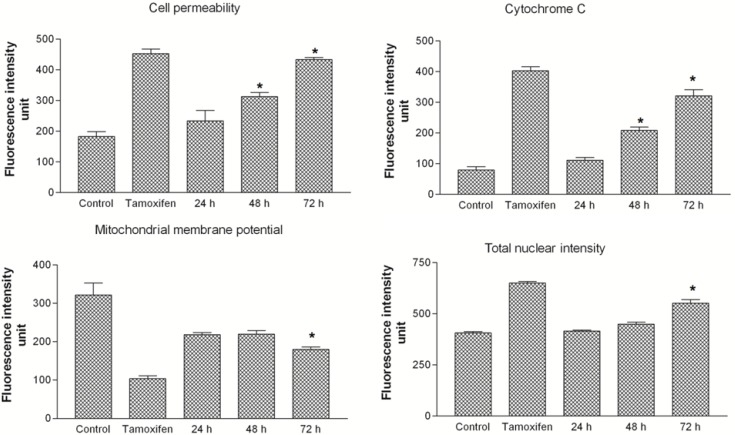
The analysis of TPHE mediated apoptosis markers. Changes in cell membrane permeability, cytochrome *c* localization, MMP and total nuclear intensity were all quantified simultaneously in MCF7 cells. Treatment with TPHE caused a significant elevation in cell membrane permeability, nuclear intensity and cytochrome *c* release associated with loss of MMP. The data are shown as the mean ± SD (*n* = 3). * represents significant difference (*p* < 0.05) compared with the control.

The elevated nuclear intensity of MCF7 cells was concurrent with marked increase in the cell membrane permeability, presented in green fluorescence. Mitochondrial membrane potential (MMP) was markedly reduced in the MCF7 cells treated with TPHE (Figure 8). Changes of MMP in MCF7 cells treated with TPHE at IC_50_ concentration for 24, 48 and 72 h showed a noticeable decrease in red fluorescence intensity (Figure 8), which confirmed the MMP collapse. In addition, TPHE treatment triggered the cytochrome *c* leakage from the mitochondria into the cytosol, which is shown by an elevation in cyan fluorescence intensity (Figure 8). The quantitative assessment of respective apoptotic changes in MCF7 cells suggested the involvement of mitochondria-mediated apoptosis (Figure 9).

### 2.8. TPHE Induced Up-Regulation of Bax and Down-Regulation of Bcl-2 Assessed by RT-PCR and Immunofluorescent Analysis

**Figure 10:**
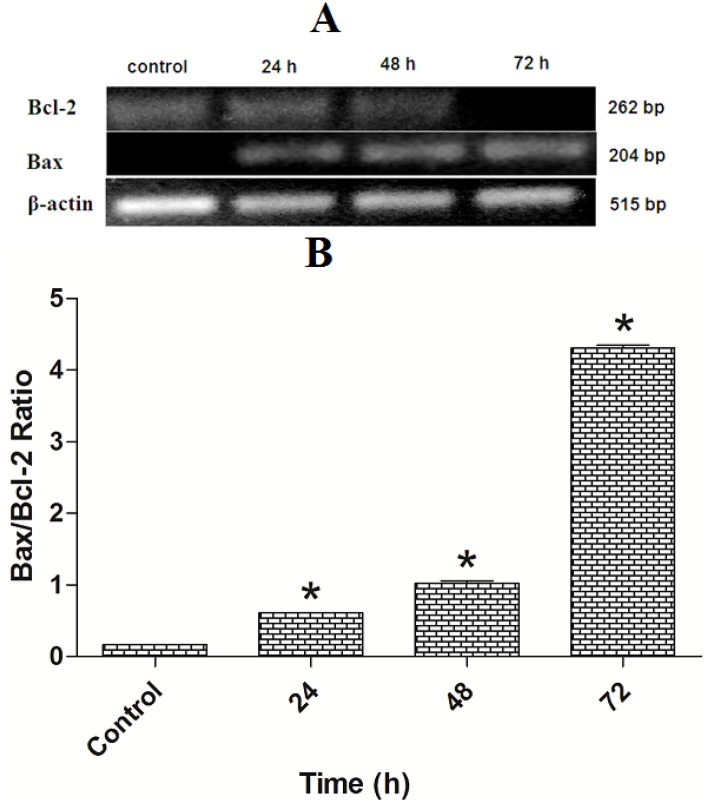
Effect of TPHE on mRNA expression of Bax and Bcl-2 in MCF7 cells. (**A**) Expression of Bcl-2 and Bax compared to β-actin in MCF7 cell line after treatment with TPHE shown in gel electrophoresis. (**B**) Time-dependent effect of THPE on Bax/Bcl-2 ratio in MCF-7 cells showed significant changes after 24, 48 and 72 h. The data are shown as the mean ± SD (*n* = 3). * represents significant difference (*p* < 0.05) compared with the control.

The expression levels of Bax and Bcl-2 mRNA were measured by RT-PCR analysis. The expression of Bax was up-regulated in treated MCF7 cells, meanwhile, the Bcl-2 expression was down-regulated after 24, 48 and 72 h treatment (Figure 10A). The Bax/Bcl-2 ratio in treated MCF7 cells showed more than a fourfold change after 72 h treatment (Figure 10B). 

**Figure 11:**
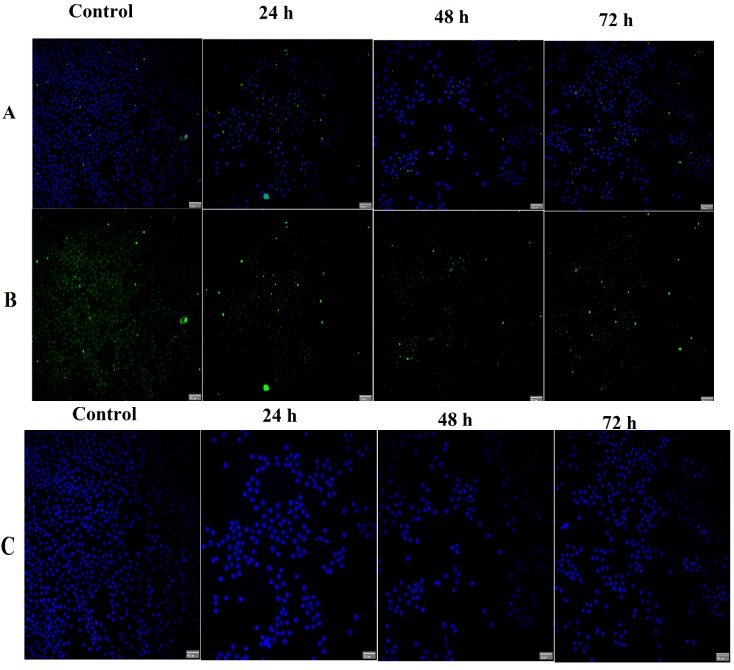
Immunofluorescent detection of Bcl-2 in MCF7 cells. The experiment was performed twice for this protein. Data revealed a decrease in the level of Bcl-2 protein after treatment. (**A**) Composite image of FITC with DAPI, (**B**) FITC conjugated with Bcl-2 and (**C**) DAPI.

**Figure 12:**
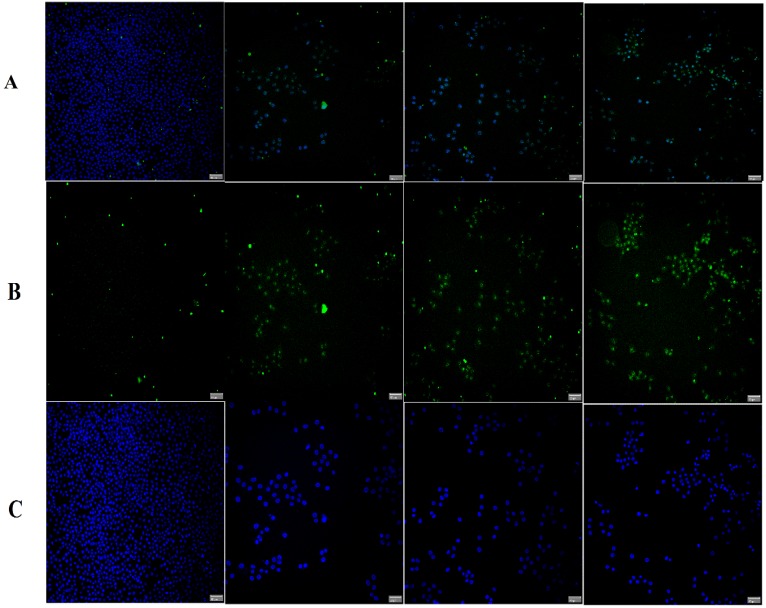
Immunofluorescent detection of Bax in MCF7 cells. The experiment was performed twice for this protein. Data revealed an increase in the level of Bax protein after treatment. (**A**) Composite image of FITC with DAPI, (**B**) FITC conjugated with Bax and (**C**) DAPI.

**Figure 13:**
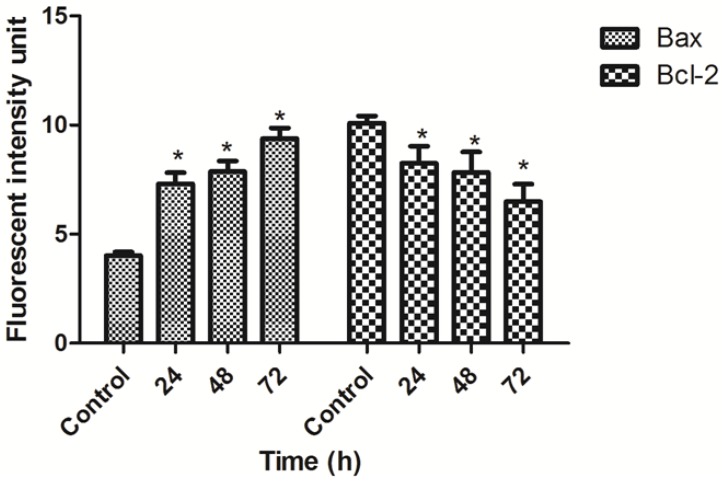
Quantitative analysis of Bax and Bcl-2 expression after treatment with TPHE in 24, 48 and 72 h. Histograms show the significant increase in the Bax as well as significant decrease in Bcl-2 ratio at protein level. The data are shown as the mean ± SD (*n* = 3). * represents significant difference (*p* < 0.05) compared with the control.

In order to confirm RT-PCR results at the protein level, the Immunofluorescence assay was performed twice for each protein. As shown in Figure 11, intensities of Bcl-2 staining immunofluorescence were decreased in treated cells compared to control. Meanwhile, intensities of Bax staining were greater in treated compared to control (Figure 12). Quantitative analysis revealed a significant increase of Bax in treated cells with TPHE associated with a significant decrease in Bcl-2 at protein level (Figure 13).

### 2.9. Inhibitory Effect of TPHE on the Migration and Invasion of MCF7 Cells

**Figure 14:**
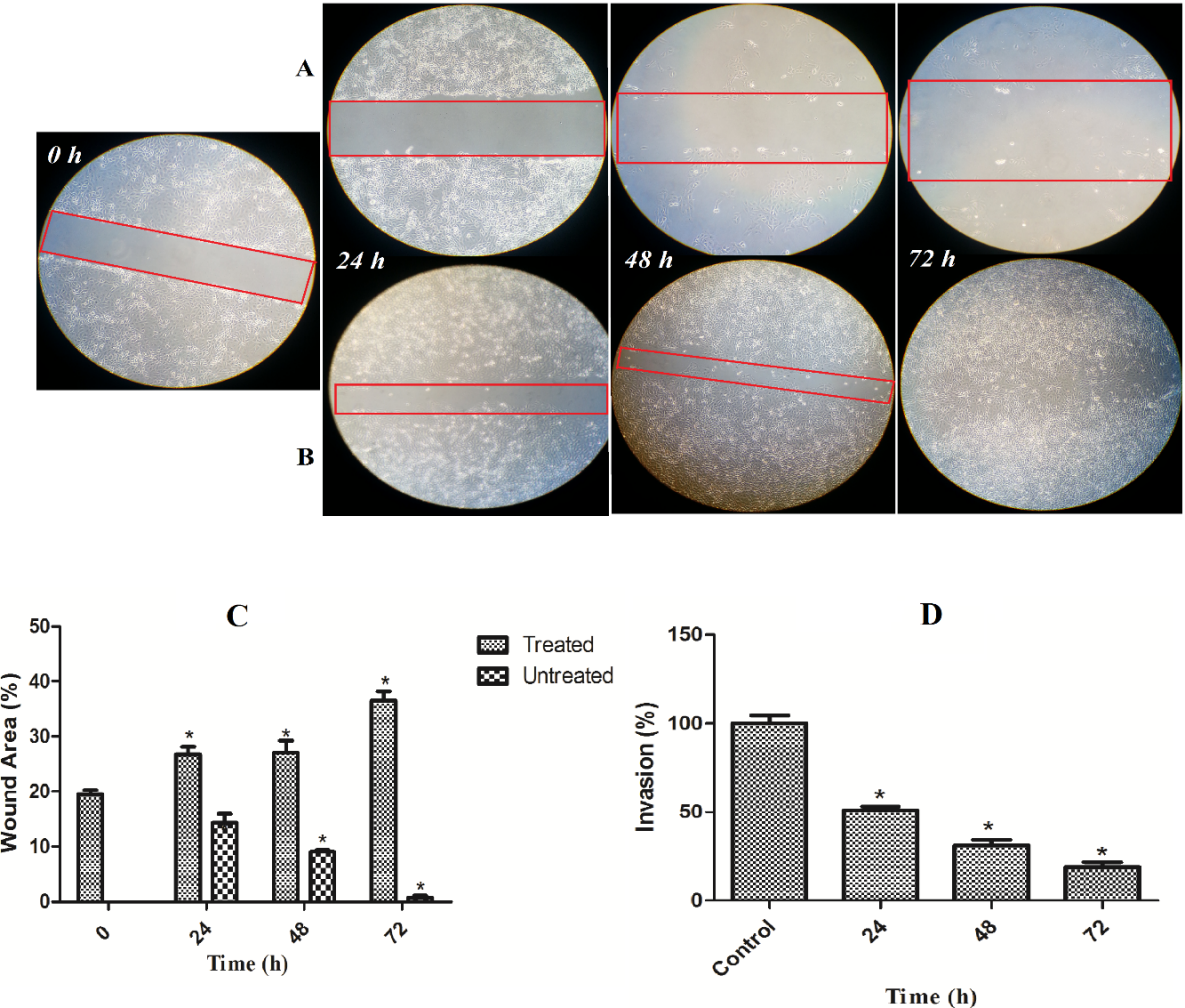
TPHE blocked MCF7 cell migration and invasion. (**A**) TPHE suppressed breast cancer cell migration. MCF7 cells were seeded in 6-well plates. The confluence MCF7 cells were wounded and imaged (0 h). After treatment of MCF7 cells with TPHE at IC_50_ concentration, cells were incubated at 37 °C for 24, 48 and 72 h and photographed. (**B**) The untreated cells of MCF7 were significantly migrated within 72 h. (**C**) The quantitative analysis of images revealed the significant suppressive effect of TPHE against MCF7 migration. (**D**) TPHE significantly suppressed MCF7 cell invasion. Percentage of invasion was determined as the percent of MCF7 cells invaded through Matrigel inserts *vs*. the total MCF7 cells invaded through the control inserts. The data are shown as the mean ± SD (*n* = 3). * represents significant difference (*p* < 0.05) compared with the control.

To determine the activity of TPHE on the metastatic potential of MCF7 cells, we investigated the TPHE inhibitory effect on migration and invasion of MCF7 cells which are markedly invasive. The result demonstrated significant inhibitory potential of TPHE against the migration of MCF7 cells (Figure 14A). In quantitative analysis, the migration capacity reduced by 40% in TPHE treated cells in comparison with control cells (Figure 14C). To explore TPHE’s potential on suppression of invasion, a chamber assay was used to measure the number of invaded cells through the barrier. As shown in Figure 14D, after treatment with TPHE, in 24, 48 and 72 h, approximately 60, 45 and 25% of MCF7 cells invaded through the barrier compared to the control, respectively. Thereby, this result implies that TPHE may have an inhibitory effect on MCF7 cell invasion (Figure 14D).

### 2.10. Discussion

Natural product-derived compounds isolated from different sources, namely marine organisms, micro-organisms and plants have been a vital source for numerous clinically useful anti-cancer agents. Plant-derived compounds with clinically anti-cancer applications, including vincristine, vinblastine, the camptothecin derivative, irinotecan, topotecan and paclitaxel (Taxol^®^) have highlighted the role of natural products in the development of new pharmaceutical agents [23]. The GC-MS-TOF analysis in our study showed that the major compound detected in TPHE is a sesquiterpene lactone. Previous studies also showed that a great number of sesquiterpene lactones were isolated from the Asteraceae family [24,25,26]. It is well established that sesquiterpene lactones are a rich group of compounds generally of plant origin, which were shown to have noticeable ani-cancer and anti-tumor activities [27,28]. One such a example is parthenolide isolated from *Tanacetum parthenium* (feverfew), which was shown to have apoptotic effects against different cancer cell lines [29,30,31]. Parthenolide showed anti-cancer effects on human acute myelogenous leukemia stem and progenitor cells through reactive oxygen species generation, pro-apoptotic activation of p53 and inhibitory effect on NF-κB [18].

The apoptotic properties of cells are the crucial key of the most approaches used in chemotherapy and radiation therapy; therefore, it has apparent therapeutic implications [32,33]. Apoptosis consists of various biochemical and morphological changes in the cells, which includes chromatin condensation, cell membrane blebbing, DNA fragmentation, mitochondrial membrane potential changes and activation of the caspase cascade [34]. In the present study, we examined the *in vitro* effect of TPHE against the MCF7 breast cancer cell line. We have found out that *Tanacetum polycephalum* (L.) Schultz-Bip has suppressive effects against different cancer cell lines, including A549, HepG2, HT29, MCF7 and MDA-MB231. The MTT assay showed that TPHE had a wide range of cytotoxic activities against these cancer cells (Figure 1), in which the IC_50_ for MCF7 cells was found to be the lowest. Thus, we focused on investigating the potential of TPHE on induction of apoptosis and its underlying mechanisms of action on MCF7 cells. It is interesting to note that the non-tumorigenic cell lines (WRL-68 and CCD841) were not affected markedly by the TPHE-mediated antiproliferative effect. Microscopic analysis using AO/PI dual staining demonstrated that following treatment with TPHE, the number of viable MCF7 cells was decreased in a time-dependent manner. Furthermore, critical apoptotic markers in treated MCF7 cells, including chromatin condensation, cell membrane blebbing were observed. These findings suggested the induction of apoptosis in MCF7 cells by TPHE, although further evidence is needed to confirm this.

Programed cell death has two distinct phases, namely early and late [35]. In healthy and viable cells, phosphatidylserine is located on the inner surface of the plasma membrane, which will translocate to the outer surface after induction of apoptosis, apparently through an active mechanism [36]. Since PS exposure is a widespread event during apoptosis that occurs earlier than DNA-associated changes and membrane leakage, Annexin-V-FITC is a probe which has been established as an easy method for detection of apoptosis in this phase [37]. Therefore, we treated MCF7 cells with the IC_50_ concentration of TPHE for 48 h, followed by supplementation with Annexin-V conjugated to FITC that could be observed by fluorescence microscope. The analysis demonstrated noteworthy PS externalization upon treatment with THPE, confirming the presence of cells at the early stage of apoptosis. In addition, as illustrated in Annexin-V-FITC flow cytometry analysis, it was found out that the number of apoptotic MCF7 cells was higher at earlier stages of the treatments. However, the number of necrotic cells was elevated at the later stage of treatment, which was possibly due to the prolonged incubation with THPE resulting in the progression of apoptosis to necrosis [38,39].

The complex cascade of caspases is a hallmark in the process of apoptosis, as it regulates the final demise of the cell [40]. Here, the levels of caspases-8 and -9 were found to increase when MCF7 cells were treated with TPHE. The activation of caspase-9 after 48 h provided evidence that TPHE was capable of triggering apoptosis via the mitochondrial pathway [41]. Moreover, the increased activity of caspase-8 level upon treatment with TPHE shed light into the possible involvement of extrinsic pathway [42]. The significant increase in the level of caspase-7 upon treatment with TPHE revealed the involvement of this executioner caspase in the induction of apoptosis. It is well established that more than one pathway usually involves in the apoptosis exerted by anticancer compounds, especially from plant derived natural sources [43,44,45]. In our research, we have observed that untreated cells showed no increase in the level of caspase-8 and -9. However, it was significantly and dose dependently increased upon treatment. Caspases activity is a critical factor in the induction of apoptotic cell death [46].

Since the activation of caspase-9 has been observed, we then examined other factors which are closely associated with mitochondria. Hence, the complex role of mitochondria in MCF7 cells was examined by the investigation of changes in MMP, as it is well established that perturbations in MMP triggers the formation of mitochondrial membrane transition pores (MPTP) [47]. The High Content Screening (HSC) analysis performed in this research showed that TPHE caused MMP loss and subsequent relocalization of cytochrome *c* from mitochondria to the cytosol. The leakage of pro-apoptotic proteins such as cytochrome *c*, the serine protease HtrA2/Omi and Smac/DIABLO is triggered due to the MMP changes and subsequent MPTP formation [48,49]. In the intrinsic pathway, the cytochrome *c* release is one of the necessary components for the formation of apoptosome and further activation of caspases [50]. The release of cytochrome *c* and the activation of caspase by TPHE clearly showed that the induced apoptosis is through the mitochondrial pathway. There is evidence that cytochrome *c* leakage is mediated by the members of the Bcl-2 protein family as the key mediators for the translocation of pro-apoptotic proteins from the intermembrane space of mitochondria into the cytosol [51,52]. Therefore, we check the expression of Bax and Bcl-2 genes to confirm the involvement of mitochondria which follows the intrinsic pathway. Down-regulation of Bcl-2 and up-regulation of Bax at the gene level after 24, 48 and 72 h confirmed that the apoptosis induced by TPHE is clearly dependent on the involvement of Bax and Bcl-2 regulation at the translational level. We again confirmed this phenomenon at the protein level by demonstrating the expression of Bax and Bcl-2. Immunofluorescence imaging was carried out and it clearly demonstrated the increase in the level of Bax compared to the down-regulation of Bcl-2 in treated cells.

The regulation of cell death and cell proliferation is tightly controlled by molecules that sometimes have a common role in the death and cell division in multicellular organisms [53]. Indeed, the process of apoptosis is regulated by the proteins that are often involved in cell cycle regulation [54]. In order to achieve this connection, we analyzed the cell cycle to confirm the involvement of apoptosis in MCF7 cells upon treatment with TPHE in a time-dependent pattern for 24, 48 and 72 h. It was found that TPHE induced cell cycle arrest at G_1_ phase following by increasing the number of cells from S and G_2_/M to G_0_/G_1_ phase. The significant elevation in the number of cell death associated with mentioned apoptotic hallmarks and factors proved the type of cell death as the apoptotic programmed death.

Metastasis as one of the hallmarks for malignant tumor cells is defined as the ability of cancer cells to spread over the adjacent organs in the human body [55]. Penetration of cancer cells into the vessel walls in another site can lead to proliferation and eventually formation of another tumor in other organs [56]. Thus, suppression of cancer cell metastasis is one of the key factors in cancer therapy. In the present study, we investigated the suppressive ability of TPHE against invasion and migration of aggressive cancer cell line, MCF7. Our result implies that TPHE has promising anti-metastatic activity against tumor cancer cells.

## 3. Experimental

### 3.1. Plant Materials

*T. Polycephalum* (L.) Schultz-Bip was collected from Shahrekord, Chaharmahalo Bakhtiari, Iran in May 2013 and identified by Dr. Mehran Karimian. A voucher specimen of this plant has been deposited at the Herbarium, Biological Institute, Shahrekord Azad University. The plant was cut into thin slices and air-dried at 25–27 °C. The dried leaves materials were ground with a mill grinder into coarse powder.

### 3.2. Preparation of Extracts from T. Polycephalum (L.) Schultz-Bip

Two and a half kilos of *T. polycephalum* (L.) Schultz-Bip were extracted at room temperature (25–27 °C) successively with analytical grade hexane, chloroform and methanol. The air-dried and powdered leaves of *T. polycephalum* were subjected to the extraction with hexane in conical flasks at the beginning. The filtrates were collected and the residues were brought to dryness and extracted with chloroform following with the same method of extraction for methanol. Soaking in increasing polarities of solvents resulted in different crude extracts were concentrated using a rotary evaporator at 45–50 °C under reduced pressure and brought to complete dryness.

### 3.3. Cell Culture and Cell Viability Assay

A549 (human lung cancer cells), CCD841 (normal human colon epithelial cells), CEMss (human T4-lymphoblastoid cells), HepG2 (human hepatoma cells), HT29 (human colon cancer cells), MCF7 (human breast cancer cells), MDA-MB-231 (human breast cancer cells), PC3 (human prostate cancer cells) and WRL-68 (human hepatic cells) cell lines were purchased from American Type Cell Collection (ATCC, Manassas, VA, USA). All the cells were maintained in RPMI-1640 (Sigma, St. Louis, MO, USA) or DMEM (Sigma) media, supplemented with 10% FBS (PAA, Pasching, Austria) and 1% penicillin/streptomycin (PAA) at 37 °C incubator with 5% CO_2_ saturation. Untreated medium containing vehicle DMSO (0.1%) was used as a vehicle control in every assay.

Viability assay was performed using MTT assay. Briefly, cells (5 × 10^4^ cells/mL) were treated with all three extracts at different concentrations (1.56, 3.12, 6.25, 12.5, 25, 50 and 100 µg/mL) in 96-well plate and incubated for 48 h. Tamoxifen (Sigma) as a positive control was tested towards the most sensitive cell line (MCF7). The colorimetric assay is examined at the absorbance of 570 nm using a microplate reader (Asys UVM340, Eugendorf, Austria). The potency of the antiproliferative effect of extracts was expressed as IC_50_ value. As *T. Polycephalum* hexane extract (TPHE) demonstrated the lowest IC_50_ value against breast cancer MCF7 cells, we used only TPHE to continue this study against MCF7 cells.

### 3.4. Chemical Analysis of TPHE

The analysis of the hexane extract was performed using an Agilent and LECO RESTEK, Rxi-5MS capillary column (30 m, 0.25 mm i.d., 0.25 µm film thickness) and a Pegasus HT High Throughput TOFMS mass spectrometer, as previously described in detail by Daferera and colleagues [57]. The carrier gas was helium at a flow rate of 1 mL/min. Column temperature was initially 40 °C for 5 min, then gradually elevated to 160 °C at 4 °C/min, and finally increased to 280 °C at 5 °C /min and hold for 10 min. For GC-MS detection, an electron ionization system was used with ionization energy of 70 eV. The fraction was diluted 1:100 (v/v) with ethyl acetate and 1.0 µL of the diluted sample was injected automatically in splitless mode. Injector temperature was set at 250 °C. Compounds were identified from their mass spectra, by comparison of the retention times of peaks with interpretation of MS fragmentation patterns from data library.

### 3.5. Acridine Orange/Propidium Iodide (AO/PI) Dual Staining Assay

Morphological changes in treated MCF7 cells were characterized using AO/PI double staining assay. MCF7 cells were cultured in 25 cm^2^ flask and incubated for 24 h. Then, Cells were treated with IC_50_ concentration of TPHE for 24, 48 and 72 h. After the incubation time, treated and untreated cells were harvested and washed twice with PBS. The pellets were stained with 5 µL of AO (1 mg/mL) and 5 µL of PI (1 mg/mL). Within 30 min, the stained MCF7 cells were analyzed under a UV-fluorescence microscope (Olympus BX51, Tokyo, Japan).

### 3.6. Annexin-V-FITC Assay

Annexin-V as a phospholipid-binding protein has a high affinity for externalized PS. Therefore, the detection of PS on the outer leaflet of cell membranes with Annexin-V serves as an easy marker for early apoptotic cells [58]. The effect of TPHE on apoptosis was analyzed in MCF7 cells. The cells (5 × 10^4^ cells/mL) were seeded into a chamber slide plate. After 48 h of TPHE treatment, they were washed in PBS and re-suspended in staining solution containing fluorescein-labeled Annexin-V-FITC and binding buffer. The cells were monitored under a fluorescence microscope (Olympus BX51).

The induction of early and late apoptosis by TPHE was further studied via an Annexin-V-FITC/PI staining assay. Briefly, MCF7 cells (1 × 10^6^) were plated in a 60-mm^2^ culture dish and treated with TPHE at IC_50_ concentration for 24, 48 and 72 h. The adherent and suspended cells were harvested and washed twice with PBS. Then, the MCF7 cells were then re-suspended in Annexin-V binding buffer (BD Biosciences, San Jose, CA, USA) and stained with Annexin-V-FITC (BD) and PI (Sigma) according to the vendor’s instructions. The fluorescent intensity of MCF7 cells was then examined using flow cytometry (BD FACSCanto™ II) and quadrant statistics for necrotic and apoptotic cell populations. Detection of early and late apoptosis was done by Annexin-V, while PI was responsible for the detection of late apoptosis and necrosis.

### 3.7. Cell Cycle Assay

MCF-7 cells in the exponential phase of growth were treated with TPHE for 24, 48 and 72 h, then harvested by trypsinization, and washed twice with ice-cold PBS and fixed by 70% ethanol (700 µL) at 20 °C for at least 30 min. The fixed cells were then washed again with ice-cold PBS and stained with 50 g/mL of PI in the presence of 100 g/mL RNase-A for 30 min. RNase-A by degradation of RNA allowed PI to bind specifically to the DNA content of MCF7 cells. Cell cycle distribution was analyzed using a flow cytometer (BD FACSCanto™ II). Data from 10,000 cells per sample were collected and analyzed using the Cell Fit Cell analysis program.

### 3.8. Caspase Analysis

The analysis of caspase-7, -8 and -9 activation upon treatment with TPHE was examined in white-walled 96-well plates using the commercial kit of Caspase-Glo^®^-7, -8 and -9 assays (Promega Corporation, Madison, WI, USA). MCF7 cells (20,000 cells/well) in the exponential phase of growth were treated with IC_50_ concentration of TPHE for 3, 6, 12, 24, 48 h. After the incubation time, the treated and untreated MCF7 cells (vehicle control) were supplemented with Caspase-Glo^®^ Reagents (50 µL). The white-walled 96-well plate was gently shaken using a plate shaker at 400–600 rpm for 20 s and incubated at 25 °C for 30 min in the dark. The induced activation of tested caspases was measured using the luminescence microplate reader (Infinite M200PRO, Tecan, Männedorf, Switzerland).

### 3.9. Multiple Cytotoxicity Assay

Multiple cytotoxicity assay was carried out using the Cellomics Multiparameter Cytotoxicity 3 kit (Cellomics, Pittsburgh, PA, USA) as described in detail previously [59]. This kit enables a single cell to be simultaneously measured for critical independent parameters of apoptosis, including changes in cell membrane permeability, cytochrome *c* release, mitochondrial membrane potential (MMP) and nuclear intensity. The stained and fixed cells of MCF7 were analyzed using the Arrayscan HCS system (Cellomics).

### 3.10. Analysis of mRNA Expression of Bax and Bcl-2 by RT-PCR

After treatment of MCF7 cells with TPHE for 24, 48 and 72 h, the commercial kit of RNeasy Mini Kit (Qiagen, Hilden, Germany) was applied to extract the total RNA from the MCF7 cells, according to the manufacture’s instruction. The reverse transcription reaction for the isolated RNA was carried out using the QuantiTect Rev Transcription Kit (Qiagen). The transcribed cDNA (1 μL) was applied for the RT-PCR amplification, with specific primers for Bax (sense, 5′-TTCTGACGGCAACTTCAACTG-3′ and antisense, 5′-AGCACTCCCGCCACAAAGA-3′), Bcl-2 (sense, 5′-AGACATCAGCATGGCTCAAA-3′, and antisense, 5′-GATTCACTGGGTAAGACTAAAGGA-3′) and β-actin (sense, 5′-CGGGAAATCGTGCGTGAC-3′, and antisense, 5′-GCCTAGAAGCATTTGCGGTG-3′) genes. β-actin gene served as the loading control in this study. RT-PCR amplification was carried out using thermal cycle. In brief, the reaction was initiated with a denaturation at 95 °C for 5 min. Amplification of DNA was continued for 30 cycles of denaturation, annealing and extension at 95 °C for 30 s, 60 °C for 40 s, and 72 °C for 1 min, respectively. The reaction was terminated after final extension at 72 °C for 10 min. The RT-PCR products were subjected to 1.2% agarose gel electrophoresis and stained with Novel Juice Cat No. LD001-1000. They were observed under UV light using the Gel Doc XR system (Bio-Rad, Hercules, CA, USA).

### 3.11. Immunofluorescence Analysis of Bax/Bcl-2

To confirm the perturbation in the activity of Bax and Blc-2 at the protein level, we carried out immunofluorescence analysis using CellReporter™ Molecular Devices (Molecular Devices, Sunnyvale, CA, USA). The MCF7 cells (5 × 10^4^ cells/mL) in the exponential phase of growth were treated with TPHE and incubated for 24, 48 and 72 h. After washing the cells twice with PBS, they were fixed in 4% paraformaldehyde at 25 °C for 15 min. Then, the cells were washed again three times with PBS and treated with blocking buffer prior to incubation in 0.03% Triton X-100/PBS and normal serum for 1 h. The fixed cells were washed again with PBS and supplemented with a diluted primary antibody solution containing 1× PBS/1% BSA/0.3% Triton X-100. The MCF7 cells were incubated at 4 °C for 24 h. Then, Bcl-2 and Bax fluorochrome-conjugated secondary antibody diluted (Santa Cruz Biotechnology, Santa Cruz, CA, USA) in antibody dilution were supplemented to the MCF7 cells and incubated for 1 h. After washing three times in PBS, cells were treated with DAPI prior to being examined using CellReporter™ Molecular Devices.

### 3.12. Migration Assay

MCF7 (2 × 10^5^ cells/well) were plated in a 6-well plate for 24 h. Then, they were scratched with a pipette tip and treated with TPHE at IC_50_ concentration. After washing the cells twice to remove nonadherent cells, photomicrographs were captured at 0, 24, 48 and 72 h after wounding using a microscope (Olympus BX51).

### 3.13. Invasion Assay

The invasion assay was done using the commercial kit of Cultrex^®^ 96 Well BME Cell Invasion Assay (Trevigen, Gaithersburg, MD, USA), according to the manufacturer’s instructions. In brief, 8 µm polycarbonate nucleopore filters (Corning Costar, Cambridge, MA, USA) were used to evenly coat a 96-well unit with 100 µL of basement membrane extract coating solution at 37 °C for 5 h. Then, MCF7 cell suspensions (2 × 10^5^ cells/mL) were placed in the upper compartment, with RPMI-1640 medium in the lower part. After treatment with TPHE for 24, 48 and 72 h, MCF7 cells that had not invaded were discarded using a cotton swab. Cells that had invaded the lower compartment were examined using a microplate reader (Tecan Infinite M200PRO).

### 3.14. Statistical Analysis

Each test was performed in triplicate. Statistical analysis was performed by SPSS-17.0 package (IBM Corporation, Armonk, NY, USA) and GraphPad prism (version 4.0 Graphpad software Inc, San Diego, CA, USA). Analyses of variance were performed using the one-way ANOVA test (Dunnett’s Multiple comparison test). The results were expressed as the mean value ± SD.

## 4. Conclusions

*Tanacetum polycephalum* (L.) Schultz-Bip has been proved for the first time to possess anticancer potential against MCF7 breast cancer cells. It was found out that TPHE has the capability of inducing mitochondrial mediated apoptosis, which was well regulated by caspase enzymes. Moreover, the active role of mitochondria in the cell death was confirmed by reducing the MMP, release of cytochrome *c* and Bax/Bcl-2 regulation. The cell cycle arrest in G_1_ phase also has been found in our study. Our results demonstrate that *Tanacetum polycephalum* (L.) Schultz-Bip is a promising anti-cancer plant. However, further research with a bioassay guided approach will need to characterize the effect of potentially anti-cancer compounds isolated from this plant. Investigation on sesquiterpene lactones isolated from this plant and their detailed mechanism of action on different cancer cell lines is also required. In addition, *in vivo* studies and clinical trials on the plant’s phytochemicals is another area of research that is vital for the development of new pharmaceuticals drugs from sesquiterpene lactones.
